# Descriptive Anatomy of the Porcine Ventral Abdominal Wall as a Basis for Training Ventral Hernia Repair Techniques

**DOI:** 10.3389/jaws.2024.12359

**Published:** 2024-03-18

**Authors:** Maaike Vierstraete, Nicky Van Der Vekens, Roel Beckers, Yohann Renard, Filip Muysoms

**Affiliations:** ^1^ AZ Maria Middelares, Ghent, Belgium; ^2^ General and Digestive Surgery, Reims University, Reims, France

**Keywords:** training model, anatomy, porcine model, ventral hernia, training

## Abstract

**Background:** In recent times there has been a surge in innovative techniques concerning complex abdominal wall surgery. The availability of simulation models for comprehensive training and skill set development remains limited.

**Methods:** Cadaveric dissections of the porcine abdominal wall were conducted to assess the suitability of anesthetized porcine models for training in both minimally invasive and open surgical procedures.

**Results:** The panniculus carnosus, a typical muscular layer in mammals, is the outermost layer covering the anterolateral abdominal wall. Beneath it, there are four main pairs of abdominal wall muscles, mirroring the human anatomy. The rectus abdominis muscle runs straight along the linea alba and is surrounded by the rectus sheath, which is formed by the fusion of the lateral abdominal wall muscles and differs along the different regions of abdominal wall. The orientation of the muscle fibers in the lateral abdominal wall muscles, i.e., musculus obliquus externus, internus and transversus, is comparable to human anatomy. Although the transition lines between their muscular and aponeurotic part differ to some extent. Relevant for the adoption of surgical techniques, the transversus abdominis muscle is well-developed and resembles a bell curve shape as it transitions from its muscular to aponeurotic part.

**Conclusion:** Despite minor differences in abdominal wall anatomy between pigs and humans, the porcine model provides a high level of fidelity in terms of both anatomical features and the development of skill sets relevant to hernia surgery.

## Introduction

A comprehensive understanding of the anatomy of the abdominal wall is crucial to successfully perform complex abdominal wall reconstructions in humans. To ensure the safety of patients and allow surgeons to develop their skills, it is essential to provide adequate training in abdominal wall surgery using simulation-based models. Previous studies have mentioned the use of porcine simulation models for training hernia repair techniques, but there is a lack of detailed information about the anatomical features of the porcine abdominal wall [1–3]. To fully assess the suitability of the porcine model as a training tool for ventral hernia repair, it is essential to have a clear understanding of its structural anatomy and how it differs from the human anatomy. The existing descriptions of porcine anatomy found in veterinary literature do not provide sufficient detail regarding the distinct layers of the abdominal wall, which are essential for designing effective training models. Therefore, the purpose of this article is to present a comprehensive description of the porcine abdominal wall anatomy, serving as a valuable basis for the development of dedicated hernia training exercises.

## Materials and Methods

Cadaveric dissections of the porcine abdominal wall were conducted using porcine cadavers utilized for laparoscopic training in hernia surgery. At the end of these training sessions, the cadavers were dissected after euthanasia, ensuring compliance with the Belgian Animal Use Act, and ethical approval was obtained under the reference EC2021/067. The pigs selected for the study were crossbreeds between Landras and Large White, aged between 14 and 16 weeks, with an approximate weight of 50 kg.

The dissections were performed collaboratively by a senior general surgeon specialized in abdominal wall surgery (FM) and a surgical resident (MV). The pig cadavers were positioned supine on the table, with their backs resting on the surface and their legs elevated. The dissection procedure began by making an incision from the xiphoid process to the pubic region, followed by a gradual removal of the skin, subcutaneous fat, and muscles on the right and left sides of the abdomen, allowing for the exposure of the distinct layers within the abdominal wall. Whenever a layer of muscle was removed, illustrative photographs were captured for reference. Additionally, the right and left sides of the abdomen were sectioned to provide an axial view of the abdominal wall. Axial T2 weighted images (Turbo Spin Echo, slice thickness 4 mm, echo time 60 msec, NSA3, ETL 19, Phase encoding steps 212) of the porcine cadaver were performed in supine position from the lower abdomen to the thoracoabdominal junction, using a 3 T magnetic resonance scanner (Ingenia 3T, Philips Medical System Best Netherlands).

## Results

### The Abdominal Wall

**FIGURE 1:**
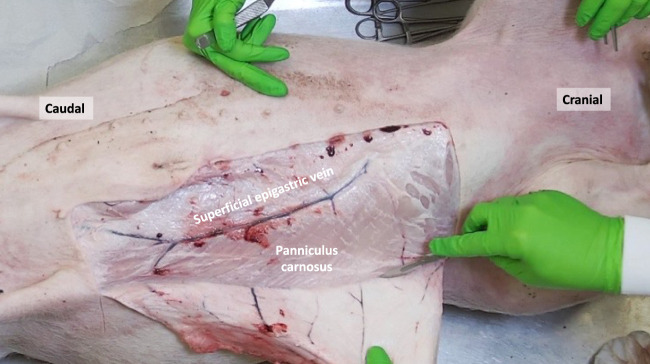
The porcine panniculus carnosus (PC) and superficial epigastric veins are exposed after removal of the skin and subcutaneous tissue.

**FIGURE 2:**
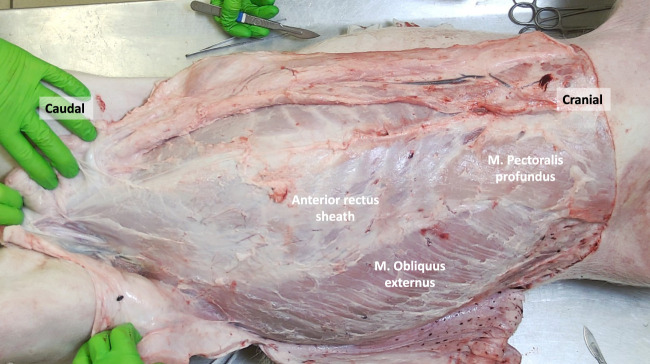
The porcine abdominal wall after removal of the panniculus carnosus, revealing the M. Pectoralis profundus and M. Obliquus externus. The aponeurotic part of the M. Obliquus externus contributes to the anterior rectus sheath.

The structural composition of the porcine abdominal wall exhibits variations across the different regions of the abdomen. The anterolateral abdominal wall consists of four main pairs of abdominal wall muscles, namely, the external oblique muscles, internal oblique muscles, transversus abdominis muscles, and rectus abdominis muscles. Progressing from the outermost layer to the innermost, the abdominal wall comprises the skin, subcutaneous tissue, panniculus carnosus, and the four pairs of abdominal wall muscles, each consisting of both muscular and aponeurotic components (Figure 1). Additionally, certain areas of the abdominal wall are covered by muscles originating from the thorax. For instance, the musculus pectoralis profundus, a deep ascending pectoral muscle, arising from the sternum and the cartilages of the first four ribs (Figure 2).

### Panniculus Carnosus

The panniculus carnosus (PC), also known as cutaneous trunci or subcutaneous muscle, is an extensive layer of striated muscle that covers the anterolateral and dorsal regions of the abdominal and thoracic wall. Its muscle fibers follow a craniocaudal and mediodorsal course and converge ventrally on the midline, where they unite with the fibers from the opposite side (Figure 1). This muscle is responsible for tensing and twitching the skin, earning it the nickname “fly shaker.” In male pigs, the PC forms the preputial muscle around the prepuce, while in female pigs, it includes the supramammary muscles that extend from the xiphoid process to the pubic region, providing coverage for the mammary glands. While the PC is mostly absent in humans, there are descriptions of different muscles at scattered anatomical locations that are considered remnants of the PC [4]. Some authors have also referred to the human superficial fascia, such as the fascia of Scarpa in the lower abdominal wall, as an evolutionary form of the PC [5]. Concerning the dermal vascularization, the superficial epigastric veins were observed to be located just lateral to the nipples. Consistent with the findings of Minqiang et al., distinct superficial epigastric arteries could not be isolated, although their existence has been mentioned in previous reports [6].

### Musculus Pectoralis Profundus

**FIGURE 3:**
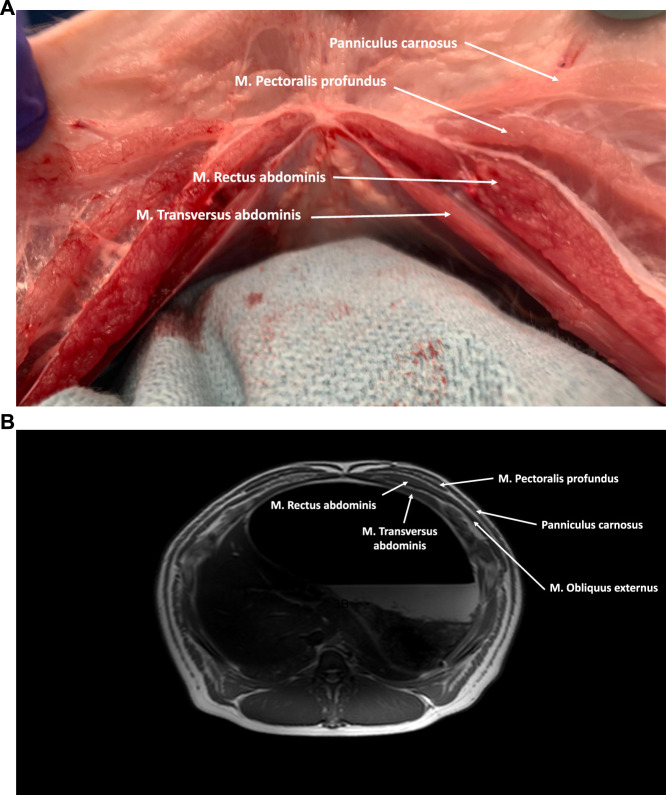
**(A)** Axial cross-section of the cranial part of the porcine abdominal wall. The M. Rectus abdominis is covered anteriorly by an aponeurotic anterior rectus sheath (M. Obliquus externus and anterior lamina of the M. Obliquus internus) and posteriorly by a musculoaponeurotic sheath (posterior lamina of the M. Obliquus internus and M. Transversus Abdominis). Anterior of the anterior rectus sheath the M. Pectoralis profundus and the panniculus carnosus can be visualized. **(B)** MRI axial cross-section of the cranial part of the porcine abdominal wall.

Once the PC is removed, the Musculus Pectoralis Profundus becomes visible, covering the medial part of the costal margin and upper segments of the Rectus Abdominis Muscle. Its muscle fibers have a craniocaudal and lateromedial direction, in parallel with the Musculus Obliquus Externus muscle fibers (Figures 2, 3).

### Rectus Abdominis Muscle

**FIGURE 4:**
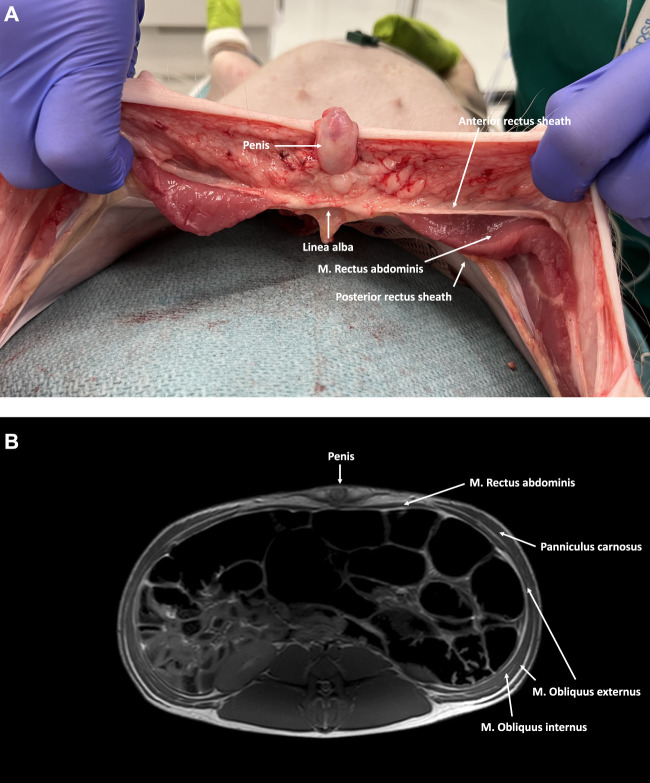
**(A)** Axial cross-section of the caudal part of the abdominal wall in a male pig. The M. Rectus abdominis is covered by an aponeurotic sheath, both anteriorly and posteriorly. **(B)** MRI axial cross-section of the caudal part of the abdominal wall in a male pig. The M. Rectus abdominis is covered anteriorly by an aponeurotic anterior rectus sheath formed by all three lateral abdominal wall muscles. No posterior rectus sheath is present below the level of the arcuate line.

The rectus abdominis muscle is a straight muscle located in the anterior abdominal wall, running parallel to the linea alba. Its origin spans from the lateral border of the sternum to the third costal cartilage, and it inserts into the prepubic tendon. Within each muscle belly, there are typically eight to ten tendinous intersections, which are transverse bands of fibrous tissue. These intersections adhere to the overlying anterior rectus sheath. The rectus abdominis muscle is surrounded by an aponeurosis, known as the rectus sheath, which is formed by the fibers of the three lateral abdominal wall muscles. Similar to humans, the composition of the rectus sheath varies in the upper and lower regions of the abdomen (Figures 3, 4).

**FIGURE 5:**
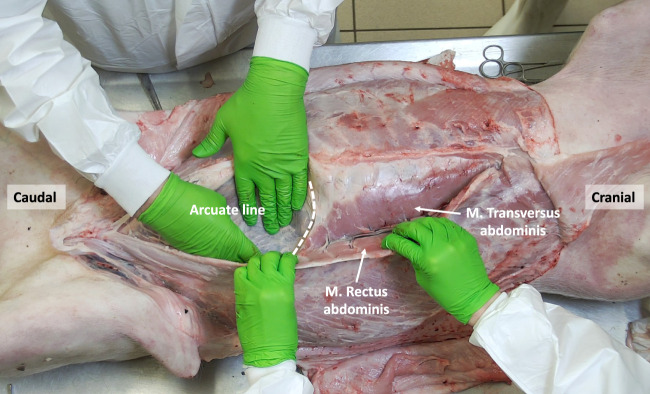
The porcine abdominal wall after mobilizing the M. Rectus abdominis laterally, showing the overlap between the M. Rectus abdominis and the underlying M. Transversus abdominis. The arcuate line designating the inferior border of the posterior rectus sheath is shown.

Cranially, the ventral aspect of the rectus abdominis muscle is covered by the anterior rectus sheath, which is formed by the fusion of the aponeuroses of the two oblique muscles (i.e., external oblique muscle and anterior lamina of the internal oblique muscle). In the lower part of the abdominal wall, the anterior rectus sheath is supplemented by the aponeurosis of the transversus abdominis muscle. The posterior rectus sheath is comprised of the posterior lamina of the internal oblique muscle and the muscular part of the transversus abdominis muscle cranially. Caudal to the level of the umbilicus, the three aponeuroses of the lateral abdominal wall muscles merge to form the anterior rectus sheath, leaving the rectus muscle covered posteriorly by the transversalis fascia only [7]. Like in humans, this demarcation point is called the arcuate line, designating the inferior border of the posterior leaf of the rectus sheath (Figure 5). The anterior and posterior rectus sheaths fuse at the level of the midline to form the linea alba, a fibrous structure extending from the xiphoid process to the pubic symphysis. The umbilicus is located approximately at the midpoint of this line and is characterized by a deep umbilical dimple on its inner side. Notably, the underlying peritoneum in the peri-umbilical region and the ventral abdominal wall is thicker in pigs compared to humans.

### External Oblique Muscle

The external oblique muscle is the most superficial among the flat anterolateral abdominal wall muscles (Figure 2). Its fibers originate from the lateral surfaces of the ribs, starting from the fourth rib as well as the fascia covering the external intercostal muscles and the lumbodorsal fascia. The muscle fibers run in a caudal-medial direction and exhibit a significant fleshy portion that covers a considerable part of the flank. As the muscle approaches the medial region, its fibers transform into an aponeurotic sheath. This sheath passes superficially over the rectus abdominis muscle, contributing to the formation of the anterior rectus sheath and the linea alba in the midline.

### Internal Oblique Muscle

**FIGURE 6:**
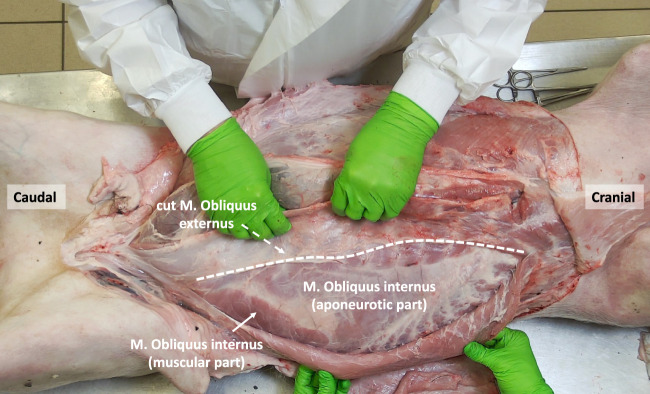
The porcine abdominal wall after mobilizing the M. Obliquus externus laterally, exposing the small muscular component of the M. Obliquus Internus as well as its larger aponeurotic part (anterior of the underlying M. Transversus abdominis).

The internal oblique muscle is located beneath the external oblique muscle and originates from the tuber coxae and the lateral portion of the inguinal ligament. At the hip region, the internal oblique muscle exhibits a relatively small yet robust muscular portion, with its fibers running in a cranial-anterior direction, perpendicular to the fibers of the external oblique muscle (Figure 6). Medially, the muscle fibers transform into a larger aponeurotic segment, which inserts into the cartilages of the last four or five ribs. In the upper part of the abdomen, the fibers of the internal oblique muscle encompass the rectus sheath both anteriorly and posteriorly by respectively its anterior and posterior lamina. However, in the lower part of the abdomen, its fibers only cross anteriorly to the rectus abdominis muscle.

### Transversus Abdominis Muscle

**FIGURE 7:**
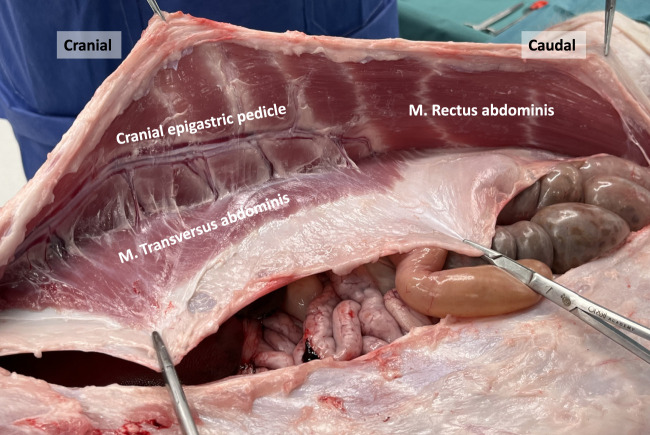
Dissection of the anterior abdominal wall in a porcine model. The posterior rectus sheath is composed of an aponeurotic and muscular part, the latter originating from the M. Transversus abdominis. The deep cranial epigastric pedicle along the inner surface of the M. rectus abdominis is visualized, as well as the neurovascular bundles.

**FIGURE 8:**
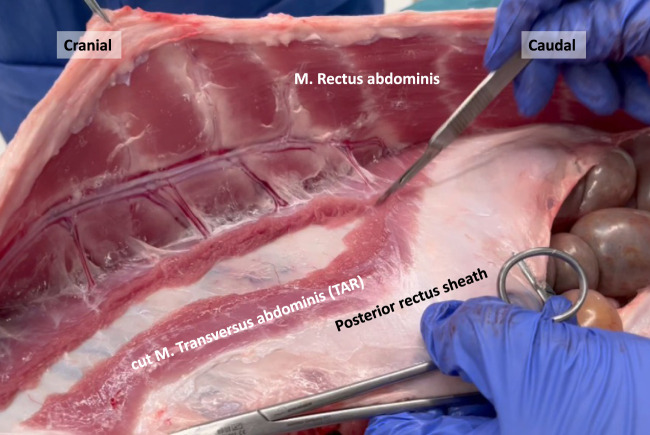
Transection of the M. Transversus abdominis resembling an open Transversus Abdominis Release (TAR) in a porcine model.

The transversus abdominis (TA) muscle is the deepest among the three anterolateral abdominal wall muscles. True to its name, its fibers run horizontally, perpendicular to the linea alba, and it possesses a well-developed muscular portion. The origin of its fibers is from the cartilages of the asternal ribs, where they meet the costal attachment of the diaphragm, as well as the transverse processes of the lumbar vertebrae. In the cranial part of the abdomen, the TA muscle fuses with the aponeurotic posterior lamina of the internal oblique muscle to create the posterior rectus sheath. In the caudal part of the abdomen, the aponeurotic segment of the TA muscle fuses with the aponeuroses of the oblique muscles to form the anterior rectus sheath. During dissection, when the rectus abdominis muscle is mobilized, the muscular portion of the TA muscle appears, lying behind the rectus abdominis muscle (Figure 7, 8). This muscle overlap between the rectus abdominis and TA is particularly pronounced in the upper part of the abdomen, where the muscular TA comes closer to the midline as it approaches the xiphoid.

**FIGURE 9:**
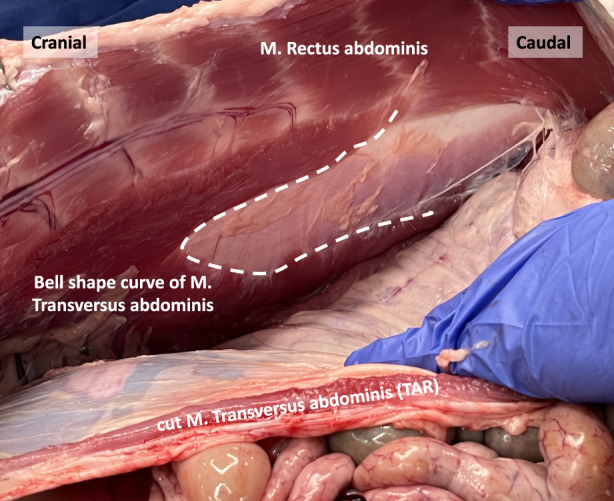
The bell shape curve of the M. Transversus abdominis after performing a Transversus Abdominis Release (TAR) in a porcine model.

In human anatomy, the transition line between the muscular and aponeurotic part of the TA muscle is known as the semilunar line. However, in our pig dissections, this line does not have a semilunar configuration. Instead, it resembles a bell curve shape, as depicted in the image obtained after performing a transversus abdominis release (TAR) procedure (Figure 9).

### Epigastric Vascular Pedicles

Plastic surgeons have previously examined the vascular supply of the porcine abdominal wall to evaluate its suitability for free-flap reconstruction training [6, 8, 9]. Unlike in humans, the primary blood supply to the anterolateral abdominal wall in pigs originates from a prominent deep cranial epigastric pedicle (Figure 7). This pedicle descends along the inner surface of the rectus abdominis muscle and divides into a medial and lateral branch at the level of the tendinous intersections. These branches resemble the neurovascular bundles found in humans and serve as important anatomical landmarks during TAR procedures. On the other hand, the deep caudal epigastric pedicle follows a shorter path and is smaller in size as it ascends along the medial aspect of the rectus abdominis muscle. There was no clear evidence of an anastomosis between the deep cranial and caudal epigastric pedicles in our observations.

## Discussion

In the realm of abdominal wall surgery, numerous innovative techniques have emerged in recent times. However, the availability of simulation models for comprehensive training and skill acquisition remains limited. To explore the validity of using anesthetized porcine models for both minimally invasive and open surgical approaches, it is essential to possess a thorough understanding of the porcine anatomy and how it diverges from human anatomy. In this regard, we investigated the anatomy of the porcine abdominal wall anatomy using cadaveric dissections. These findings serve as a basis for developing a comprehensive set of training exercises specifically tailored to ventral abdominal wall surgery.

To begin with, our observations revealed a thicker peritoneum in both the peri-umbilical region and the ventral abdominal wall of pigs, as compared to humans. This anatomical difference implies that a preperitoneal ventral hernia repair, referred to as ventral TAPP (transabdominal preperitoneal), might be relatively easier to perform in the porcine model. Consequently, the challenges associated with maintaining the peritoneal integrity during a preperitoneal dissection of the ventral abdominal wall in humans may not be accurately reflected.

Furthermore, the presence of the PC in the subcutaneous plane of the pig adds complexity to the process of optical trocar entry for an extraperitoneal surgical approach. In case of a lateral eTEP (enhanced totally extraperitoneal) approach an additional muscular layer must be traversed to accurately reach the desired retrorectus space. When employing a superior eTEP approach starting on the rib cage, as currently suggested by some practitioners, the musculus pectoralis profundus constitutes a second additional muscular layer that must be counted during optical entry. These additional muscular layers should not confuse the surgeons when opening the midline because neither the PC, nor the musculus pectoralis profundus cross the midline anterior to the linea alba.

Moreover, there is a distinctive configuration of the TA muscle in pigs, characterized by a broader and more prominent muscular component. This results in more overlap between the TA muscle and rectus abdominis muscle in the upper part of the abdomen, compared to human anatomy. Consequently, in a transabdominal lateral retrorectus (TARUP) approach, the incision of the ipsilateral posterior rectus fascia requires the division of a greater number of TA muscular fibers to access the retrorectus space. In humans, such muscular transections typically occur only at the upper edge of the posterior rectus fascia opening. Similarly, in the case of a posterior component separation by TAR, a larger quantity of muscular fibers needs to be cut, particularly in the upper abdominal region near the xyphoid, where the muscular part of the TA muscle even extends towards the midline.

Apart from slight differences in the abdominal wall anatomy between pigs and humans, we are convinced that the pig model offers a remarkably realistic and accurate training experience for hernia surgery. Besides performing procedure specific dissections, the use of an anesthetized pig allows for the practice of certain surgical actions, such as achieving precise hemostasis of bleeding vessels and managing muscle contraction during dissection. These crucial skills cannot be effectively trained in human cadavers. Additionally, gaining access to human cadavers is difficult, and comes at a high cost. Based on our current understanding, the pig model therefore exhibits a high level of fidelity in terms of both anatomical features and the development of skill sets relevant to hernia surgery.

## Data Availability

The original contributions presented in the study are included in the article/supplementary material, further inquiries can be directed to the corresponding author.
